# Supplementary material for: From sequence to enzyme mechanism using multi-label machine learning
Source: BMC Bioinformatics. 2014 May 19;15:150. doi: 10.1186/1471-2105-15-150 (PMC4229970; doi:10.1186/1471-2105-15-150)
Supplement: Additional file 2 — Java code of ml2db. Additional file ml2db_code.tar.gz contains the Java source code to run the multi-label machine learning experiments and save the results to database. The code’s Javadoc is included. [file 1471-2105-15-150-S2.zip › additional file 2/ml2db/ml2db/doc/test/mulan/mulanarff/package-summary.html]

test.mulan.mulanarff


---


|  |  |  |
| --- | --- | --- |
| **Overview** \| **Package** \| Class \| **Use** \| **Tree** \| **Deprecated** \| **Index** \| **Help** | |  |
| **PREV PACKAGE**   **NEXT PACKAGE** | **FRAMES**    **NO FRAMES**     **All Classes** |  |


---

## Package test.mulan.mulanarff

| **Class Summary** | |
| --- | --- |
| **IdInstanceTest** | Class |
| **IdMultiLabelInstancesTest** | Class |
| **MulanArffTest** | Class |
| **MulanDataSetDbLoaderTest** | Class |
| **MulanDataSetGeneratorTest** | Class |
| **MulanDataSetManagerTest** | Class |
| **MulanDataSetWriterTest** | Class |
| **MulanInstancesFillerTest** | Class |
| **SerializerTest** | Class |
| **StatisticsTest** | Class |

---


|  |  |  |
| --- | --- | --- |
| **Overview** \| **Package** \| Class \| **Use** \| **Tree** \| **Deprecated** \| **Index** \| **Help** | |  |
| **PREV PACKAGE**   **NEXT PACKAGE** | **FRAMES**    **NO FRAMES**     **All Classes** |  |


---
